# The Development and Validation of the Indian Family Violence and Control Scale

**DOI:** 10.1371/journal.pone.0148120

**Published:** 2016-01-29

**Authors:** Ameeta S. Kalokhe, Rob Stephenson, Mary E. Kelley, Kristin L. Dunkle, Anuradha Paranjape, Vikram Solas, Latika Karve, Carlos del Rio, Seema Sahay

**Affiliations:** 1 Emory University School of Medicine, Department of Medicine, Division of Infectious Diseases, Atlanta, Georgia, United States of America; 2 Emory University Rollins School of Public Health, Department of Global Health, Atlanta, Georgia, United States of America; 3 University of Michigan School of Nursing, Department of Health Behavior and Biological Sciences, Ann Arbor, MI, United States of America; 4 Emory University Rollins School of Public Health, Department of Biostatistics and Bioinformatics, Atlanta, Georgia, United States of America; 5 South African Medical Research Council, Gender and Health Research Unit, Pretoria, South Africa; 6 Temple University School of Medicine, Department of Medicine, Section of General Internal Medicine, Philadelphia, Pennsylvania, United States of America; 7 Department of Social & Behavioral Research, National AIDS Research Institute, Pune, Maharashtra, India; Medical University of Vienna, AUSTRIA

## Abstract

The high prevalence of domestic violence (DV) among married women in India and associated negative health repercussions highlight the need for effective prevention strategies and tools to measure the efficacy of such interventions. Literature supporting differing manifestations of DV by culture underscores the need for a culturally-tailored scale to more effectively measure DV in the Indian context. We therefore aimed to develop and validate such a tool, the Indian Family Violence and Control Scale (IFVCS), through a mixed-methods study. The psychometric development of IFVCS is herein discussed. After field pre-testing and expert review, a 63-item questionnaire was administered to a random sample of 630 married women from May-July 2013 in Pune, India. The item response theory approach for binary data to explore the IFVCS structure suggested that IFVCS is reliable, with the majority of items having high (>0.5) and significant factor loadings. Concurrent validity, assessed by comparing responses to IFVCS with the validated, abridged Conflict Tactics Scale-2, was high (r = 0.899, p<0.001) as was the construct validity, demonstrated by its significant association with several established DV correlates. Therefore, initial assessment of the IFVCS psychometric properties suggests that it is an effective tool for measuring DV among married women in India and speaks to its capacity for enhancing understanding of DV epidemiology and for evaluating the effectiveness of future DV interventions.

## Introduction

According to the Indian National Family Health Survey-3 (NFHS-3), two out of five married women in India report experiencing physical, sexual, or psychological abuse from their intimate partners[1]. Those who report experiencing intimate partner violence (IPV) have higher frequencies of mental health disorders[2–5], sexually transmitted infections including HIV[6–8], injuries[9], and non-communicable diseases including malnutrition[10], asthma[11], and gynecologic disorders[8,12]. The negative health effects of IPV are not only restricted to the survivor; rather, her children are also more likely to suffer from malnourishment[10], lower vaccine coverage[13], and asthma[11], and to die early[14–16]. Therefore, the high prevalence at which IPV occurs in India and its associated toll on familial morbidity and mortality emphasizes the need for effective IPV assessment and prevention strategies.

Our review of the past decade of Indian literature demonstrates wide variance in reported frequencies of marital or domestic violence (DV). For example, the median lifetime frequencies of psychological, physical, and sexual abuse reported by Indian women were 22%, 29%, and 12%, respectively, but ranged from 2–99%, 2–99%, and 0–75%, respectively[17]. The substantial variation in responses can in part be attributed to differences in the populations surveyed. Differences may also stem from unwelcome, yet controllable, sources of variation such as interviewer bias, perceived safety of the respondent during the interview, interview methodologies employed [18], the multitude of forms and perpetrators of abuse surveyed, inconsistencies in defining what constitutes DV[19], and the types of screening instruments used. Such unintentional variance not only contributes to inaccurate prevalence estimates, but may also impact our capacity to identify and measure outcomes and risk factors of DV, and hinder systematic cross-study comparisons. Validated scales have infrequently been employed in Indian DV literature; furthermore, the scales that have been used (i.e. Index of Spouse Abuse[4], Conflict Tactics Scale (CTS) used in the NFHS-3[20], the Abuse Assessment Screen[21], Composite Abuse Scale[22], the Domestic Violence Questionnaire[23], and Partner Violence Screen[24]), were largely developed in vastly different cultural contexts and seldom adapted for present-day India. To the best of our knowledge, over the past decade only one measure, the Domestic Violence Questionnaire, has in part been developed and validated in India[23]. This instrument, however, was largely an adaptation of the CTS and WorldSAFE questionnaires. Furthermore, it restricted its surveillance of DV to that perpetrated by intimate partners rather than that perpetrated by other members of the marital family. Additionally, the generalizability of the tool is questionable as it was adapted using a very specific population (women from the highly-developed and educated state of Kerala who were employed in banks or engaged in a governmental financial empowerment program). Lastly, the Domestic Violence Questionnaire minimally surveys many forms of control and psychological forms and its construct and concurrent validity have undergone limited evaluation.

Although DV is experienced by women across the world, culture in part shapes how it manifests[25]. Our prior qualitative work[19] and review of the literature[17] suggest that while some aspects of DV suffered by married women in India are similar to those reported by women in other countries, many aspects also differ. For example, women in India may encounter psychological abuse in the form of insults for infertility[26–28], for not bearing male children[5,29,30], and for insufficient provision of dowry at the time of marriage[27,31–36]. Control over reproductive decision-making, mobility, finances, access to food, and medical care are often encountered [37–40]. Guns are rarely the tools used to perpetrate physical abuse, rather more readily-available tools such as belts, sticks, broomsticks, rolling pins, pestles and chemical- or fire-induced burns are used[19,31,41,42].Our qualitative studies suggest that forms of sexual abuse appear to be influenced by affluence, wherein men with substantial disposable income may be more likely to buy pornography and force their wives to replicate sex acts against their will [19]. And, the commonly-encountered patrilineal joint family structure in which women cohabitate with their spouses and marital family post-marriage, enables members of the marital family to play either or both a protective, mediatory role in husband-on-wife DV perpetration or to serve as DV perpetrators against women as well[43]. Lastly, experiences of DV are not static within India itself, but rather influenced by rapidly-evolving technology (i.e. stalking via social networking sites and control over mobile communications)[19]. While many of the aforementioned forms of DV exist in other countries, they may be more pronounced in specific cultural settings where gender inequities are magnified.

These differences suggest currently available scales may be inadequate for measuring DV experienced by women in India and highlight the need for developing and testing such a tool in the population of its intended use. For example, the abridged CTS-2, widely used in the India NFHS-3 to obtain national DV estimates and explore DV epidemiology[20], fails to explore DV perpetrated by non-spousal members of the marital family, to explore economic control (i.e. control over *stridhan* (dowry) or material assets given to a bride by her parents at the time of marriage), “new-age” social control of electronic communications like text messaging or social networking sites, control over reproductive decision-making, psychological abuse resulting from insufficient dowry or having girl-children, and physical abuse resulting from readily-available tools. We therefore conducted a mixed-methods study to develop and validate an instrument, hereafter entitled the “Indian Family Violence and Control Scale (IFVCS),” to more effectively measure the physical, sexual, and psychological abuse and control experienced by a married woman at the hands of her spouse and marital family in India. While the qualitative formative research is published elsewhere[19], we herein describe the findings of the pretesting and initial validation in a developmental sample of 630 married women in Pune, India.

## Methods

### Ethics Statement

This study was approved by the Ethics Committee of the National AIDS Research Institute (NARI, Pune, India) and the Emory University (Atlanta, GA, USA) Institutional Review Board. Written informed consent was obtained from all subjects prior to enrollment in the study.

### Study Design

The study employed a 3-phase mixed-method design. Phase I (September 2012-January 2013) included qualitative formative methods (reported elsewhere[19]) to inform the development of the 63-question item pool. It entailed the conduct of key informant interviews with experts in DV and family counseling and gender-concordant focus groups with lay community members to understand community perceptions of the definition of DV, perpetrators of DV, and examples of DV encountered by married women residing in Pune. Phase II was conducted between March and April 2013 and involved 1) translating the items from English into Hindi and Marathi, 2) field pre-testing the items among 10 married, female peers and behavioral study staff to identify discrepancies between the individuals’ understanding of the items and the information intended to be extracted by the items, and to identify difficulties encountered by the interviewers in administering the scale, and 3) review of the items by a team of DV experts and the institutional Ethics Committee. Feedback from both field-pretesting and the expert review was used to revise the content and phrasing of the preliminary scale. The resultant items were ordered by hypothetical domains (i.e. control, psychological, physical, and sexual abuse) based on knowledge of existing structures of a similar scale (the Conflict Tactics Scale (CTS)-2). Then, two additional scale versions were generated with random item ordering to evaluate whether item order affected response. These preliminary scales were administered to a developmental sample of 630 married women during Phase III between May and July 2013.

### Participant recruitment and enrollment

To be eligible for phase III of the study, subjects had to be 1) a married female residing with her spouse, 2) age 18 years or older, 3) fluent in either Marathi, Hindi, or English, and 4) have capacity to provide informed consent and complete a one-hour questionnaire. They were not required to be literate, because the tool was designed to be self- or interviewer-administered.

Random, geographically-clustered sampling was utilized for recruitment of subjects for the Phase III developmental sample. The Pune municipality is geographically divided into 14 wards, which were further geographically divided into five sub-wards. Approximately nine participants were recruited per sub-ward. To recruit and enroll the subjects, the study team began walking in a straight line from the center to the periphery of the ward, skipping 50 households and then recruiting a participant, skipping the next 50 households and then recruiting a participant, etc., until nine participants were enrolled in the respective sub-ward. If multiple women who met inclusion criteria were present in a household, one was selected at random (by choosing a concealed number from the interviewer’s hand).

### Data collection

Interviewers verbally administered the survey in face-to-face interviews (n = 626) with the exception of a few interviews in which the participants insisted on reading and completing the survey themselves (n = 4). The surveys were conducted in privacy in the participant’s home in the language of the participant’s choice (Hindi, Marathi, or English) and included demographic questions, the 63-item preliminary scale, and the 12-item abridged CTS-2 used in the Indian NFHS-3 (to assess construct validity). Questions for the hypothetical control domain utilized the umbrella question, “During my entire married life, without being bothered by my husband or his family, I could…,” and a 4-point Likert scale (never, rarely, sometimes, often). Questions from the hypothetical psychological, physical, and sexual violence domains utilized the umbrella question, “My husband or a member of his family…,” a one-year sampling frame, and the 5-point Likert scale (never, about once or twice in the past year, about once a month, about once a week, and not in the past year but it did happen before in my married life).

### Participant and Study Team Safety

Safety procedures were developed in line with the WHO recommendations for the conduct of DV research. The study staff members who conducted the interviews were female [44] and had prior professional experience establishing rapport and asking sensitive study questions in other research studies. They underwent extensive training in methods to optimize privacy, participant safety and comfort during the interview, and confidentiality of the data, as well as to facilitate participant referral to DV support services when necessary. For their own safety, the staff was always accompanied into the field by a male study staff member and often a neighborhood NGO worker. The male study team member remained present in the neighborhood with active mobile phone until the interview was completed and the interviewer had exited the home. To address potential interviewer emotional fatigue and distress associated with repeated DV interviewing, the study staff were informed of local support services and required to participate in a weekly team debriefing session with the study investigators.

To optimize participant safety, on approaching a household the interviewers introduced themselves as social workers from a governmental institution conducting a one-hour ‘women’s health survey,’ as is recommended by the WHO guidelines[44]. They then asked how many women met study inclusion criteria and randomly chose one subject to participate as described above. Next, they requested a private place to conduct the interview in the household given the ‘sensitive nature’ of the health questions. If a woman was unable to provide this, the study staff asked her if she would prefer a different time or venue (i.e. a nearby partner NGO site). If this was not possible, rather than run the risk of compromising the participant’s safety, the household was skipped and the adjacent household was sampled. Upon establishing a private space for the interview, the study staff explained the true intent (i.e. examination of their DV experiences) and content of the interview to the participant using the written consent form. And after obtaining informed consent, the interviewer explained that if a family member were to walk into the room during the interview that she would stop asking about DV and shift to asking physical health questions (i.e. about diabetes or heart disease). Upon completion of the interview, a debriefing session was held with each participant so they could further explain their answers and ask questions of the interviewer. Additionally, they were provided a list of community DV support services concealed in a phone diary of other community resources (i.e. grocery stores). The study staff offered to facilitate their referral to the support services.

### Statistical analysis

The sample size of 630 participants was based on expert recommendation of enrolling ten subjects per item to be tested (i.e. 63x10 = 630)[45]. The written surveys were entered into Microsoft Office Access 2007 and transferred to SPSS and STATA for further analysis. Descriptive statistics were used to analyze the demographic data and to explore frequencies of response to each individual scale item. On initial review of the item response frequencies it became apparent that several items suffered from low variance due to “zero inflation”[46], (i.e., a larger amount of none/never responses than would be expected if the underlying variable were indeed continuous). Zero inflation leads to both a deflation of the mean for continuous data analysis, and the other categories having small sample sizes which would lead to poor estimation for ordinal models. We therefore recoded the variables into binary (no, yes) using the never category as the cutoff in each case. An analysis of the correlation structure among the two versions of the variables (not shown) indicated this resulted in very little loss of variance for the item response theory (IRT) analysis. Thus, although we first conducted confirmatory factor analysis (CFA) analyzing each item as continuous and used Cronbach’s alpha as our measure of internal consistency, we repeated the CFA using an IRT model for binary data. Given this was an adaptation of an already validated scale, a series of 2-parameter IRT models were run for each subdomain (control, sexual, etc.) to test for unidimensionality within each domain. These models give an estimator of the response rate for the median individual as well as the correlation between the item and the factor (standardized factor loading), and were fit using the gllamm add-on to Stata [47]. Univariate analysis of variance and the tests of between-subjects effects were used to evaluate whether scale version (i.e. item order) or language of administration affected response. Lastly, Pearson Correlation, t-tests, and univariate analysis of variance was used to evaluate congruence in response to the scale response and demographic variables and the NFHS-3 CTS-2 questionnaire. All tests used a two-tailed alpha of 0.05.

## Results

### Pre-testing

Scale pre-testing among the ten married, female study staff and NGO peers and expert review of the items led to several modifications of the scale’s content and administration prior to use in Phase III. First, the initial 5-point and 6-point Likert scales used for the control and violence subscales, respectively, were too lengthy for the participants to recall. Second, many participants asked for further clarification on the time frame of the ‘not in the past year but it did happen to me before’ and thus ‘in my married life’ was added. And, the interviewers reported that filling circles was too time-consuming and interrupted interview flow and that increased spacing was needed between questions to ensure erroneously shading the wrong response for the wrong question. Lastly, many interviewees felt dissatisfied with being unable to explain their responses. This led to the inclusion of a debriefing session at the end of the interview for participants to expand on their responses if they chose to do so.

### Participant characteristics

A total of 630 randomly-sampled married women from 14 wards in the Pune municipality participated in Phase III (Table 1). The average age of the participants was 35 years (σ = 10 years), three quarters (78% or 491/630) were Hindu, less than half (42% or 264/630) were employed, two-fifths (39% or 247/630) completed education beyond the tenth standard, and half (47% or 298/630) had family monthly incomes of less than Rupees 10,000 per month. For the overwhelming majority, the current marriage was their first (98% or 620/630) and it had been arranged (87% or 545/630). The average age of marriage was 15 years (σ = 10). Slightly over half (58% or 363/630) resided in nuclear families in low- to mixed-quality housing (53% or 332/630). The average age of the participants’ spouses was 40 years (σ = 11), the majority (93% or 586/630) were employed, and two-fifths (44% or 277/630) completed education beyond the tenth standard. On average, participants had 2 children (σ = 1) with the average age at which the participants bared their first child being 21 (σ = 4) years. More participants (77% or 486/630) reported having a pregnancy resulting in the live birth of a male child than female child (63% or 400/630). A significant number reported having planned abortions (16% or 100/630) and miscarriages or still-births (18% or 111/630).

**Table 1:** Background Characteristics of Phase III Study Participants (married women over age 18 years in Pune, India (n = 630)).

	No. (%)
Age, mean (SD), *years*	35 (10)
Religious affiliation	
- Hindu	491 (78)
- Buddhist	77 (12)
- Muslim	46 (7)
- Other (i.e. Christian, Jain)	15 (2)
Employment status	
- Employed	264 (42)
- Not employed	366 (58)
Education level	
- No formal education	67 (11)
- ≤7^th^ standard	141 (22)
- 8-10^th^ standard	175 (28)
- ≥10^th^ standard	247 (39)
Family monthly income	
- <Rs. 6000	110 (17)
- Rs. 6000–10,000	188 (30)
- >10,000	280 (44)
- Unknown to participant	45 (7)
Individuals residing in household, mean (SD)	5 (2)
Housing material	
- Low-quality (i.e. thatch, mud)	73 (12)
- Mixed-quality (i.e. mix of low and high quality material)	259 (41)
- High-quality (i.e. cement, bricks, tiles, concrete)	298 (47)
Marital duration, mean (SD), *years*	15 (10)
Marriage	
- First	620 (98)
- Second or higher	10 (2)
Type of marriage	
- Arranged	545(87)
- Autonomous	84 (13)
Family structure	
- Nuclear	363 (58)
- Joint	263 (42)
Age of spouse, mean (SD), *years*	40 (11)
Employment status of spouse	
- Employed	586 (93)
- Not employed	43 (7)
Educational level of spouse	
- No formal education	51 (8)
- ≤7^th^ standard	118 (19)
- 8-10^th^ standard	181 (29)
- ≥10^th^ standard	277 (44)
Age at first child, mean (SD), *years*	21 (4)
Pregnancies, mean (SD)	2 (1)
Had one or more pregnancies resulting in:	
- a live-birth, male child	486 (77)
- a live-birth, female child	400 (63)
- a planned abortion	100 (16)
- a miscarriage or still-birth	111 (18)

### Internal consistency

Using the IRT analysis, standardized factor loadings were determined for each item (Table 2). The vast majority of items demonstrated good internal consistency, with only 7 of 63 items failing to reach statistical significance. The items with low factor loadings included: *“spend my own or self-earned money on my children*,*” “take up a new job or remain in my current job if I wanted to*,*” “bothered me for being infertile*,*” “tried to poison me*,*” and “purposely made me drunk or high on drugs to force me to have sex against my will*.*”* One item *“burnt me or threatened to burn me with a cigarette*,*”* was dropped from the IRT analysis due to low variance (only 1/630 responded affirmatively). The subscale scores were all significantly correlated (range of Pearson correlations 0.35 to 0.84).

**Table 2:** Factor analysis of items in the Indian Family Violence and Control Scale (n = 630) with median percent response and standardizing factor loadings of each item.

	Median percent response	Standardizedfactor loading
**Control Subscale**		
*During my entire married life*, *without being bothered by my husband or his family*, *I could…*		
- *rest and relax when I wanted to*.	.10	.71
- *spend my own or self-earned money on my natal family*.	.74	.76
- *spend my own or self-earned money on my children*.	.09	.28
- *spend my own or self-earned money on my friends*.	.65	.79
- *spend my own or self-earned money for my personal things*.	.12	.69
- *take up a new job or remain in my current job if I wanted to*.	.61	.29
- *go out of the house*.	.07	.80
- *visit my natal family*, *friends*, *coworkers*, *relatives*, *or other acquaintances*.	.11	.79
- *talk freely on the phone or send SMS (text) messages*.	.05	.82
- *seek medical care for myself*.	.01	.82
- *make my own decisions about family-planning such as getting pregnant*, *using contraception*, *spacing between children*, *and permanent sterilization*.	.19	.54
- *wear any type of dress and have any style that I wanted*.	.27	.50
- *freely invite my natal family members and friends to visit me in my matrimonial home*.	.11	.78
- *have sex how and when I wanted to*.	.21	.56
**Psychological Violence Subscale:**		
*My husband or a member of his family…*		
- *screamed at me when I was alone*.	.60	.71
- *excessively criticized me for my work at home*.	.15	.68
- *screamed at me or insulted me in front of others*, *in a public place*, *or on a social networking site*.	.24	.74
- *threatened that he/they would send me out of the house*.	.01	.90
- *forced me to leave the house*.	.01	.85
- *threatened to send me to my natal home against my will*.	< .01	.90
- *sent me to my natal home against my will*.	< .01	.91
- *harassed me for wedding-related gifts or money such as maanpaan or dowry*.	.03	.73
- *harassed my natal family for wedding-related gifts or money such as maanpaan or dowry*.	.02	.78
- *taunted me about my poor health*.	.03	.78
- *threatened to hurt or hurt my children because he/she was angry with me*.	.09	.58
- *threatened to hurt or hurt a member of my natal family because he/she was angry with me*.	.04	.77
- *threatened to leave me and get remarried*.	.02	.76
- *intentionally spread false rumors about my character and chastity*.	.02	.77
- *intentionally ignored me or did not talk to me*.	.30	.64
- *intentionally starved me or gave me stale food*.	< .01	.86
- *intentionally confined me in the house*.	< .01	.80
- *intentionally left me out of family functions or social events*.	.06	.70
- *bothered me for having a girl child*.	.01	.65
- *bothered me for being infertile*.	.02	.17 (NS)
- *forced me to become vegetarian or non-vegetarian*.	.01	.56
- *forced me to fast (perform upvas) when I did not want to*.	.01	.64
**Physical Violence Subscale**		
*My husband or a member of his family…*		
- *forced me to work excessively against my will*.	.04	.71
- *slapped or scratched me*.	.36	.91
- *kicked*, *punched*, *or beat me*.	.01	.96
- *twisted my arm or pulled my hair*.	.02	.93
- *pushed me*, *pulled me*, *dragged me*, *shook me*, *or held me down*.	.04	.89
- *tried to strangle or suffocate me*.	< .01	.87
- *tried to hang me*.	< .01	.87
- *tried to poison me*.	< .01	.45 (NS)
- *threw things in the house when he/she was angry with me*.	.14	.65
- *burnt me or threatened to burn me with a cigarette*.	*Dropped due to low variance*	*Dropped due to low variance*
- *threatened to burn me using kerosene*, *chemicals*, *acid*, *or some other method*.	< .01	.78
* -burned me using kerosene*, *chemicals*, *acid*, *or some other method*.	< .01	.76
- *threatened me with a sharp object such as broken glass*, *a razor blade*, *axe*, *or knife*.	< .01	.84
- *attacked me with a sharp object such as broken glass*, *a razor blade*, *axe*, *or knife*.	< .01	.92
- *threatened me with a blunt object such as a belt*, *stone*, *broomstick*, *or rolling pin*.	.01	.88
- *attacked me with a blunt object such as a belt*, *stone*, *broomstick*, *or rolling pin*.	< .01	.92
**Sexual Violence Subscale**		
*My husband or a member of his family…*		
- *forced me to have sex against my will during my menstrual cycle*.	.03	.71
- *forced me to have sex against my will with someone else*.	< .01	.85
- *purposely made me drunk or high on drugs to force me to have sex against my will*.	< .01	.96 (NS)
- *forced me to have sex without a condom against my will*.	< .01	.80
- *forced me to replicate a sexual behavior from a pornographic film against my will*.	< .01	.84
- *forced me to engage in vaginal sexual intercourse against my will*.	.07	.81
- *forced me to engage in oral sex against my will*.	< .01	.87
- *forced me to engage in anal sex against my will*.	< .01	.88
- *videotaped us having sex against my will*.	< .01	.57 (NS)
- *intentionally performed forceful sex to hurt me*.	< .01	.83
- *threatened to sexually abuse someone that I care about if I refused to have sex*.	< .01	.89

NS = not significant

### Concurrent and construct validity

**Fig 1:**
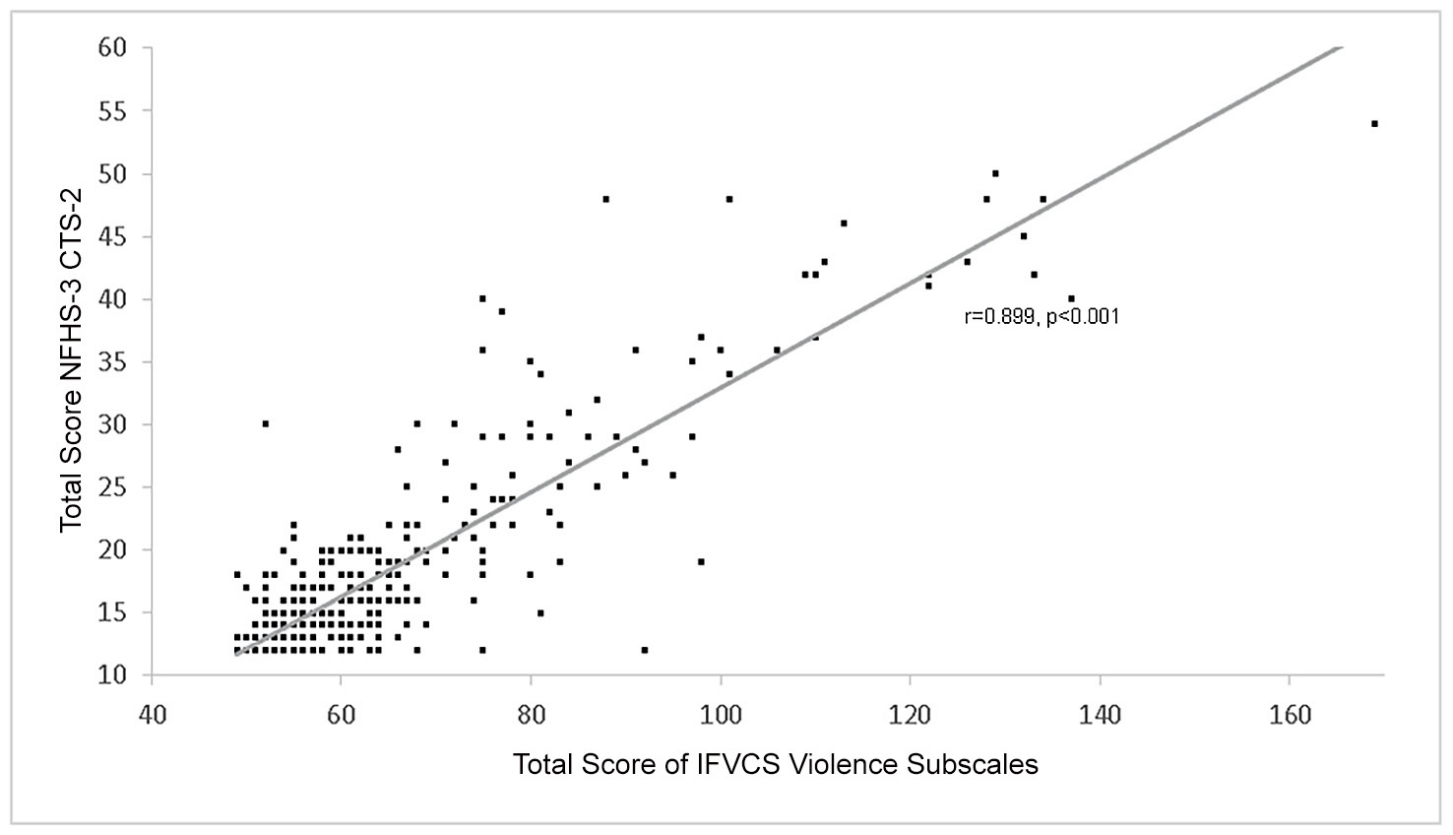
Total Score of the Indian Family Violence and Control Scale Violence Subscales and National Family Health Survey-3 Conflict Tactics Scale-2 Total Score for all participants (n = 630).

Concurrent validity was assessed by comparing IFVCS responses to the abridged 12-item CTS-2 (widely used in India to assess IPV in the NFHS-3)[20]. The Pearson correlation demonstrated a strong and significant association (r = 0.899, p<0.001) between responses to the two scales (Fig 1). Construct validity was assessed by evaluating the association between DV correlates established in the literature and the IFVCS control and violence subscales as well as the abridged CTS-2 (Table 3). The IFVCS control and violence subscales held up equally well as the NFHS-3 CTS-2 for most DV correlates (i.e. participant education level, family monthly income, spouse’s education level, number of pregnancies, age of participant when she had her first child, and quality of housing materials) and performed better than the NFHS-3 CTS-2 for some others (i.e. difference in age between participant and spouse, participant employment, fertility problems). Importantly, all variable demonstrating correlation with NFHS-3 CTS-2 were also significantly correlated with the IFVCS.

**Table 3:** Assessment of construct validity comparing IFVCS Control and Violence Subscales to established DV correlates among women (n = 630).

DV correlate	IFVCS Control Subscale	IFVCS Psychological subscale	IFVCS Physical subscale	IFVCS Sexual subscale	NFHS-3 CTS-2
Participant education level	-0.245***	-0.120**	-0.237***	-0.137***	-.155***
Family monthly income	-0.319***	-0.113**	-0.163***	-0.154***	-.242***
Number of individuals living in household	0.214***	0.051	0.099*	0.053	0.099*
Difference in age between participant and spouse	0.118**	0.081*	0.113**	0.014	0.026
Spouse’s education level	-0.254***	-0.140***	-0.243***	-0.165***	-0.210***
Number of pregnancies	0.127***	0.102**	0.185***	0.161***	0.108**
Age of participant at first child	-0.254***	-0.145***	-0.237***	-0.171***	-.199***
Participant employment (employed vs. unemployed)	t = 1.676^+^	t = -0.794	t = -1.786^+^	t = -2.556*	t = -0.882
Spouse’s employment (employed vs. unemployed)	t = -0.624	t = -0.405	t = -0.214	t = -1.055	t = -1.099
Fertility problems (present vs. absent)	t = 1.772^+^	t = 0.790	t = 0.695	t = 0.156	t = 1.047
Housing materials (low- vs. mixed- vs. high-quality)	F = 19.581***	F = 5.167**	F = 11.408***	F = 4.957**	F = 12.78***

Significant correlations are noted as follows:

^+^p<0.10

*p≤0.05

**p≤0.01

***p≤0.001

Where test statistics are not followed by p-values, the correlations were not deemed significant.

### The effect of scale order, language, and the interviewer on response

As participant fatigue, interviewer-interviewee rapport, and participant understanding have potential to fluctuate during the course of an interview, three versions of the IFVCS control and violence subscales with different item ordering were randomly administered (n = 208 version 1, n = 211 version 2, and n = 211 version 3) to assess the effect of item order on response. Univariate analysis of variance and the tests of between-subjects effects failed to demonstrate an association between item order and responses. Similar analysis was used to evaluate the association between language of survey administration (n = 5 in English, n = 29 in Hindi, and n = 596 in Marathi) and IFVCS response. Interestingly, language of administration of the survey was significantly associated with differences in IFVCS response on the control subscale (English μ = 0.80, Hindi μ = 5.97, Marathi μ = 4.85, p = .01) but not the violence subscales. Multivariate analysis including participant education level and family monthly income, demonstrated the association between language of administration and control subscale response was explained by income but not education level. Lastly, an interviewer effect on the violence and control subscales was noted, (with mean violence subscale scores for interviewers 1, 2, 3, and 4 being 5.72, 3.92, 3.96, and 6.50, respectively, p<0.001, and mean control subscale scores for interviewers 1, 2, 3, and 4 being 4.26, 3.46, 4.13, and 4.87, respectively, p < .01).

## Discussion

The IFVCS was developed to fulfill the need for a culturally-tailored instrument measuring DV experienced by married Indian women. It was designed to survey the full spectrum of abuse and control that may be perpetrated against a woman by her spouse and marital family. While substantial formative research was undertaken to optimize the IFVCS content and expert validity in Phase I and II of the study[19], Phase III was dedicated to assessing other psychometric properties including the scale’s reliability and concurrent and construct validity.

Initial assessment of the IFVCS psychometrics suggests that it is reliable and may emerge as a more accurate measure of DV in the Indian context than instruments developed in the Western context such as the CTS-2. In the IRT, the majority of items had high (>0.5) and significant factor loadings. Cumulatively, the IFVCS violence subscales demonstrated excellent concurrent validity with the heavily-used and validated, abridged CTS-2 scale (r = 0.899, p<0.001). Initial evaluation of construct validity suggests that the IFVCS may be a more accurate measure of DV in India than the abridged CTS-2, as it was significantly associated with more established DV correlates (i.e. difference in age between participant and spouse, participant employment, and fertility problems) than the CTS-2. Importantly, all established DV correlates that were significantly associated with the NFHS-3 CTS-2 were also associated with response to the IFVCS.

The strong psychometric properties of the scale and successful validation can be attributed to the thorough research methodologies that were employed. First, to ensure the IFVCS item pool embraced the full spectrum of what Pune lay community members and individuals with expertise in the fields of DV and marital counseling perceived as constituting DV, substantial formative research was conducted in Phase I. Second, the item pool underwent extensive community pre-testing and revision in Phase II prior to its large-scale validation. Third, as interviewee perceived safety is paramount to enhance the integrity of their responses (and of course, their well-being), considerable effort was dedicated to training study staff in methodologies to optimize interview confidentiality and privacy in accord with the WHO guidelines[44]. Fourth, our large sample size was adequate for the validation, and random geographic-clustered subject sampling was effective in yielding a participant population representative of Pune, India. This is apparent when comparing the Pune 2011 Census to the demographics of our study population. Additionally, the lack of effect of item order on response to the subscales, suggests that respondent fatigue did not interfere with the responses. Lastly, after controlling for income, there was no discernible effect of language of administration on scale response either. We chose to evaluate income as a potential confounder because the association between socio-economic status and DV is well-supported by the Indian literature[20] and income is associated with higher English language proficiency and use in India.

In spite of the strong study design and rigorous study methodologies, some limitations of the study are apparent. First, 5 of the 63 items had low or non-significant factor loadings. The reason for low or non-significant factor loadings for some items was low response variance for some items (i.e. 1/630 participants responded affirmatively to *‘tried to poison me*,’ 1/630 participants to *‘burnt me or threatened to burn me with a cigarette*,*’* 1/630 participants to *‘purposely made me drunk/high to force sex*,*’* and 1/630 participants to *‘videotaped us having sex without consent’*), some items not being applicable to many women (i.e. 272/630 participants did not find *‘take up a new job/remain in current job if I wanted to’* applicable and 393/630 participants did not find *‘bothered me for being infertile’ applicable*), and a combination of the two reasons for *‘spend my own money on my children’* (with 41/630 reporting this item as not applicable and 605/630 responding with ‘*often’*). The items with low discriminant capacity like other items were developed based on findings from the formative research and thus included in the initial scale. Perhaps, additional surveillance of burning with a *beedi* (a leaf-wrapped tobacco product more commonly used in India than cigarettes) would increase affirmative response frequency to the item, *‘burnt me or threatened to burn me with a cigarette*.’ While we acknowledge that they do not seem to carry much relevant information in this developmental sample, we have chosen to retain them for future testing of the scale in a larger, nationally-representative sample where levels of response may be higher. Second, while the 63 evidence-based items render the IFVCS a comprehensive, effective research tool to survey the full realm of DV, the scale length may lead to interviewee fatigue if used as part of a larger multi-question survey. Additionally, the scale was only validated in women age 18 and over, and thus needs further testing to evaluate its capacity to measure DV in child marriages. Lastly, there existed an interviewer effect on response likely attributed to differences in rapport-building and interviewing skills between study staff. While all interviewers went through the same rigorous training sessions, interviewers 1 and 4 were previously HIV counselors while interviewers 2 and 3 were prior HIV study recruitment and retention field staff. The potential for the interviewer to serve as a factor influencing response to the IFVCS needs to be taken into account in future studies that utilize the instrument.

The psychometric properties assessed in this study suggest the IFVCS is a strong tool for measuring DV among married women in Pune, India. Future research should be dedicated to assessing test-retest reliability, evaluating discriminant validity, and validating the scale in rural and tribal settings, regions in India outside of Maharashtra given the state-to-state socio-cultural diversity, and other populations (i.e. women in same-sex relationships, dating relationships, child brides, widows, and men). Furthermore, future studies should aim to validate the capacity of the IFVCS to measure DV in other regions of South Asia where DV may manifest similarly. Similarly, while the current form of the IFVCS surveys *experience* of DV, rewording and retesting of the instrument to assess DV *perpetration* is also worthwhile. Lastly, future efforts should aim to validate an abridged version of the IFVCS that could be used in clinical settings or as part of a larger scale survey questionnaire.

In conclusion, initial validation of the IFVCS suggests that it is an effective tool for measuring physical, sexual, psychological abuse and control of married women by their spouses and members of their marital families. It has capacity to enrich understanding of DV epidemiology in India and thus enhance development of culturally-tailored DV prevention strategies, and to evaluate the efficacy of such interventions in reducing DV.
